# The impact of gaming addiction on Health-Related Quality of Life in adults

**DOI:** 10.1192/j.eurpsy.2022.2129

**Published:** 2022-09-01

**Authors:** C. Neily, M. Maalej, I. Gassara, R. Feki, N. Smaoui, L. Zouari, J. Ben Thabet, S. Omri, N. Charfi

**Affiliations:** Hedi Chaker hospital, Psychiatry Department, Sfax, Tunisia

**Keywords:** Quality of Life, gaming, adults, Addiction

## Abstract

**Introduction:**

Although gaming addiction has received a great deal of attention from researchers, few studies have evaluated its effect on health related quality of life in adults

**Objectives:**

To study the relationship between gaming addiction and perceived health status

**Methods:**

We conducted a cross-sectional, descriptive and analytical study.Data were collected using a self-administered questionnaire on social networks targeting adults between 18 and 40 years. We used the gaming addiction scale (GAS) in its validated Arabic short version. we also used the 36-Item Short Form Health Survey questionnaire (SF-36) in its validated Arabic version

**Results:**

One hundred and nine participants were included. The mean age was 29.6 ±10.3. Males accounted for 60.6% of the study population. A history of anxiety or depression was found in 4.6 % of participants and 3.6% had an organic affection .The mean GAS score was 13.11± 6.08. According to this scale, 25.7% were addicted gamers. We found a significant difference between the group of participants considered addicts and those who were not in the following items: vitality (p=0.002), mental health (p=0.004) and role limitation due to emotional health (p=0.05). We found a correlation between the GAS score and role limitation due to physical problems ( p= 0.41), role limitations due to emotional problems (p=0.004 ), vitality( p=0.005) and mental health ( p= 0.001).

**Conclusions:**

Our data showed significantly lower health related quality of life related to higher exposure to games especially in the psychological health.In future researches, the effect of gaming addiction on other domains of quality of life can be investigated

**Disclosure:**

No significant relationships.

